# Forecasting imported COVID-19 cases in South Korea using mobile roaming data

**DOI:** 10.1371/journal.pone.0241466

**Published:** 2020-11-04

**Authors:** Soo Beom Choi, Insung Ahn

**Affiliations:** 1 Department of Data-centric Problem Solving Research, Korea Institute of Science and Technology Information, Daejeon, Republic of Korea; 2 Center for Convergent Research of Emerging Virus Infection, Korea Research Institute of Chemical Technology, Daejeon, Republic of Korea; South China University of Technology, CHINA

## Abstract

As the number of global coronavirus disease (COVID-19) cases increases, the number of imported cases is gradually rising. Furthermore, there is no reduction in domestic outbreaks. To assess the risks from imported COVID-19 cases in South Korea, we suggest using the daily risk score. Confirmed COVID-19 cases reported by John Hopkins University Center, roaming data collected from Korea Telecom, and the Oxford COVID-19 Government Response Tracker index were included in calculating the risk score. The risk score was highly correlated with imported COVID-19 cases after 12 days. To forecast daily imported COVID-19 cases after 12 days in South Korea, we developed prediction models using simple linear regression and autoregressive integrated moving average, including exogenous variables (ARIMAX). In the validation set, the root mean squared error of the linear regression model using the risk score was 6.2, which was lower than that of the autoregressive integrated moving average (ARIMA; 22.3) without the risk score as a reference. Correlation coefficient of ARIMAX using the risk score (0.925) was higher than that of ARIMA (0.899). A possible reason for this time lag of 12 days between imported cases and the risk score could be the delay that occurs before the effect of government policies such as closure of airports or lockdown of cities. Roaming data could help warn roaming users regarding their COVID-19 risk status and inform the national health agency of possible high-risk areas for domestic outbreaks.

## Introduction

Since coronavirus disease (COVID-19) was first reported in Wuhan in December 2019, a total of 11 451 030 confirmed cases, including 534 320 deaths, have been reported in 188 countries as of July 6, 2020 [1]. The first imported case in South Korea was reported on January 20, 2020; the traveler had arrived from Wuhan, China [2]. The number of confirmed COVID-19 cases in South Korea rapidly increased February 19, 2020 onward, and Daegu city was identified as the epicenter of regional spread [2]. To delay the spread of COVID-19 without vaccines, governments in most countries have implemented nonpharmaceutical interventions such as home isolation, social distancing, airport closures, and international travel bans [3]. In Germany, the number of daily confirmed COVID-19 cases decreased after the enforcement of lockdown but increased after the lockdown was eased [4]. To prevent another COVID-19 outbreak after the ban on international travel is relaxed, the government should consider the risks posed by imported cases as the infection rate in other countries is accelerating.

Air travel data are important factors in assessing the risk from imported cases of infectious diseases [5]. Mobile phone data can provide population travel information that can be used to estimate the risk of an epidemic [6, 7]. Mobile roaming data can be particularly useful as it can estimate near real-time air travel and determine the date and duration of stay at the destination [8]. A high volume of international airline travelers arriving from countries with many confirmed COVID-19 cases may increase the number of imported COVID-19 cases in South Korea. After the World Health Organization announced the COVID-19 pandemic, governments in most countries decided to enforce international travel bans. The International Air Transport Association announced that passenger demand in May 2020 dropped 91.3% compared to that in May 2019 [9]. Government response to COVID-19 can help estimate the risks from imported COVID-19 cases. The Oxford COVID-19 Government Response Tracker (OxCGRT) provides a systematic cross-national and cross-temporal measure to understand how government responses have evolved from January 22, 2020 to the present day [10]. A stricter government response could result in fewer imported cases of COVID-19.

In this study, we aimed to develop a risk score for COVID-19 cases imported into South Korea using global COVID-19 data, Korea Telecom (KT) roaming data, and the OxCGRT index. We used this risk score to further validate the prediction models for daily imported cases in South Korea. Our hypothesis was that the risks from imported cases are positively related to COVID-19 surveillance worldwide and the number of mobile roaming users and negatively associated with the index for International Travel Controls in OxCGRT. Information regarding such risks from imported COVID-19 cases may help roaming users from spreading infection through travel.

## Materials and methods

### Data collection

We obtained data on the daily series of imported cases of COVID-19 in South Korea from January 22, 2020 to July 12, 2020 that are publicly available from daily reports of the Korea Centers for Disease Control and Prevention (KCDC) [11]. The confirmed COVID-19 cases in South Korea are reported daily and include information concerning imported cases and local outbreaks. The imported cases were confirmed after overseas travel from China, Asia except China, Europe, America, Africa, and Oceania and were categorized as per the regions in South Korea: Seoul, Busan, Daegu, Incheon, Gwangju, Daejeon, Ulsan, Sejong, Gyeonggi-do, Gangwon-do, Chungcheongbuk-do, Chungcheongnam-do, Jeollabuk-do, Jeollanam-do, Gyeongsangbuk-do, Gyeongsangnam-do, Jeju, and Lazaretto [11]. The number of daily confirmed COVID-19 cases in other countries is reported by the John Hopkins University Center and is publicly available in the COVID-19 data repository at Github [1]. Population data were obtained from Worldometer to calculate the incidence rate of each country [12].

Mobile roaming data were supported by KT, the second-largest telecommunications service provider holding 31.6% of the market share among mobile phone service providers in South Korea in 2019 [13]. KT collaborated with the Ministry of Science and ICT and provided mobile data to research institutes to develop a prediction model on the spread of COVID-19 [14]. The research on predicting the spread of COVID-19 was conducted under the strict security management of the Data Safe Zone operated by the Ministry of Science and ICT, South Korea [15]. The Data Safe Zone is a space created within the Korea Data Agency and provides a safe and secure environment to research and analyze data of sensitive public institutions and private companies [16]. KT roaming data can only be accessed from the Data Safe Zone in Seoul, South Korea, with the approval of the Korea Data Agency. KT roaming data provide the number of daily roaming users per variable, which are fully anonymized before researchers can access them, and hence, researchers cannot access personal identifying information. For this study using KT roaming data, the author visited the Data Safe Zone four times on 05-23-2020, 06-07-2020, 06-12-2020, and 07-26-2020. The roaming data included the daily number of roaming users, hometown of users, start day of roaming service, return date, and country of travel origin. The KT roaming data from January 22, 2020 to April 30, 2020 were used at first and later updated with data up to June 30, 2020.

Data on government responses to COVID-19 were obtained from OxCGRT, which provides scores for several sections, namely “Closures and Containment,” “Economic Measures,” “Health Measures,” and “Miscellaneous” [10]. We used the “International Travel Controls” index in the “Closures and Containment” section of the OxCGRT, which records international travel restrictions. The index was scored using the following ordinal scale: no measures (0 points), screening (1 point), quarantine arrivals from high-risk regions (2 points), ban on arrivals from some regions (3 points), and ban on arrivals from all regions or total border closure (4 points) [10]. The time-series data of imported COVID-19 cases from January 22, 2020 to July 12, 2020 in South Korea were used as the output variable. The time-series data of global confirmed COVID-19 cases, KT mobile roaming data, and OxCGRT from January 22, 2020 to June 30, 2020 were used as input variables.

### Risk score

To simplify input variables and maximize efficiency, we used the global incidence rate of confirmed COVID-19 cases, KT mobile roaming data, and “International Travel Controls” index of OxCGRT to calculate the risk score from imported COVID-19 cases. The incidence rate is the number of COVID-19 cases (N_covid) divided by country-specific population (P), and the global incidence rate is the sum of the incidence rates of all countries (k).

Global incidence[t] = ∑_(k = country)_N_covid_k_ [t]/P_k_

The KT roaming data are the product of the number of roaming users (N_Roaming_) and the average of days traveled (D_Roaming_).

Roaming[t] = N_Roaming_[t] × *Average* (D_Roaming_)[t]

The value of “no measures” in the OxCGRT changed from 0 to 1. We hypothesized that the risk of imported COVID-19 cases is proportional to the incidence rate of global COVID-19 cases, the number of roaming users, and the duration of travel. Moreover, we theorized that the risk is inversely proportional to the government response index. Finally, the risk score was calculated by dividing the product of the global incidence rate and the KT roaming data by the “International Travel Controls” index of OxCGRT (Govern), as shown below.

Risk score[t] = Roaming[t] × Incidence[t]/Govern[t]

### Preprocessing

The time-series data were smoothed using a 7-day simple moving average to reduce the weekly effect: smoothed Y_t_ = (Y_t_+Y_t-1_+···+Y_t-6_)/7, wherein Y is the daily observed value and t is the time [17]. Cross-correlations were analyzed to find a time lag between output and input variables using Pearson’s correlation, with the time lag range of ± 30 days and Bonferroni’s correction [18]. The resultant time lag was used to forecast the daily imported COVID-19 cases determined after the day of the time lag with data available only at the current point [18]. The training set accounted for 66% of 100 days from January 22 to April 30, and the rest was used for the first validation. Moreover, the second validation set was from May 1 to June 30 using the updated KT roaming data.

### Statistical analyses

To forecast daily imported COVID-19 cases in South Korea, we developed prediction models using simple linear regression (LR) and autoregressive integrated moving average (ARIMA), including exogenous variables (ARIMAX). ARIMA without the risk score was included as a reference. In both LR and ARIMAX, the dependent variable was the daily imported COVID-19 cases shifted backward by the forecast day, and the independent variable was the risk score calculated using global COVID-19 cases, KT mobile roaming data, and OxCGRT index. An ARIMA model includes parameters such as *p* of the autoregressive order, *d* of the differencing, and *q* of the moving average order [18].

ARIMAX(*p*,*d*,*q*) can be represented by

ϕ(L)(1−L)^d^Y_t_ = β(L)X_t_ + θ(L)ε_t_

where ε_t_ is white noise, ϕ(L) is autoregressive polynomial, (1−ϕ_1_L−ϕ_2_L^2^−…−ϕ_p_L^p^), and θ(L) is moving average polynomial, (1+θ_1_L +…+θ_q_L^q^), where L is a lag operator, where X_t_ represents exogenous variables, β their coefficients [19].

We selected the optimal parameters by performances of the first validation set. The periodical term was investigated using autocorrelation function and partial autocorrelation factor diagrams of time-series data.

To validate the time-series forecast, we selected a rolling window analysis for the training set and included the forecast values for each trial [20]. For example, the forecast, imported COVID-19 cases after 12 days on April 1, 2020 only used the variables from January 22, 2020 till March 31, 2020 as the training set. The model forecasts imported COVID-19 cases on May 28, 2020, and this procedure was repeated daily. The correlation coefficient, *R*, was calculated by Pearson correlation analysis. Root mean square error (RMSE), mean absolute error (MAE), and mean absolute percentage error (MAPE) were calculated using real and predicted values [21–24]. The goodness of fit for the model was validated using the Akaike information criterion (AIC) index and Bayesian information criterion (BIC), and smaller values corresponded to a better model fit [25–28]. We included the Durbin–Watson statistic to detect the presence of autocorrelation in the residuals from the LR model [29]. All statistical analyses were performed using Python 3.6.2 (Python Software Foundation), and p-values < 0.05 were considered statistically significant.

## Results

### Risk score

**Fig 1:**
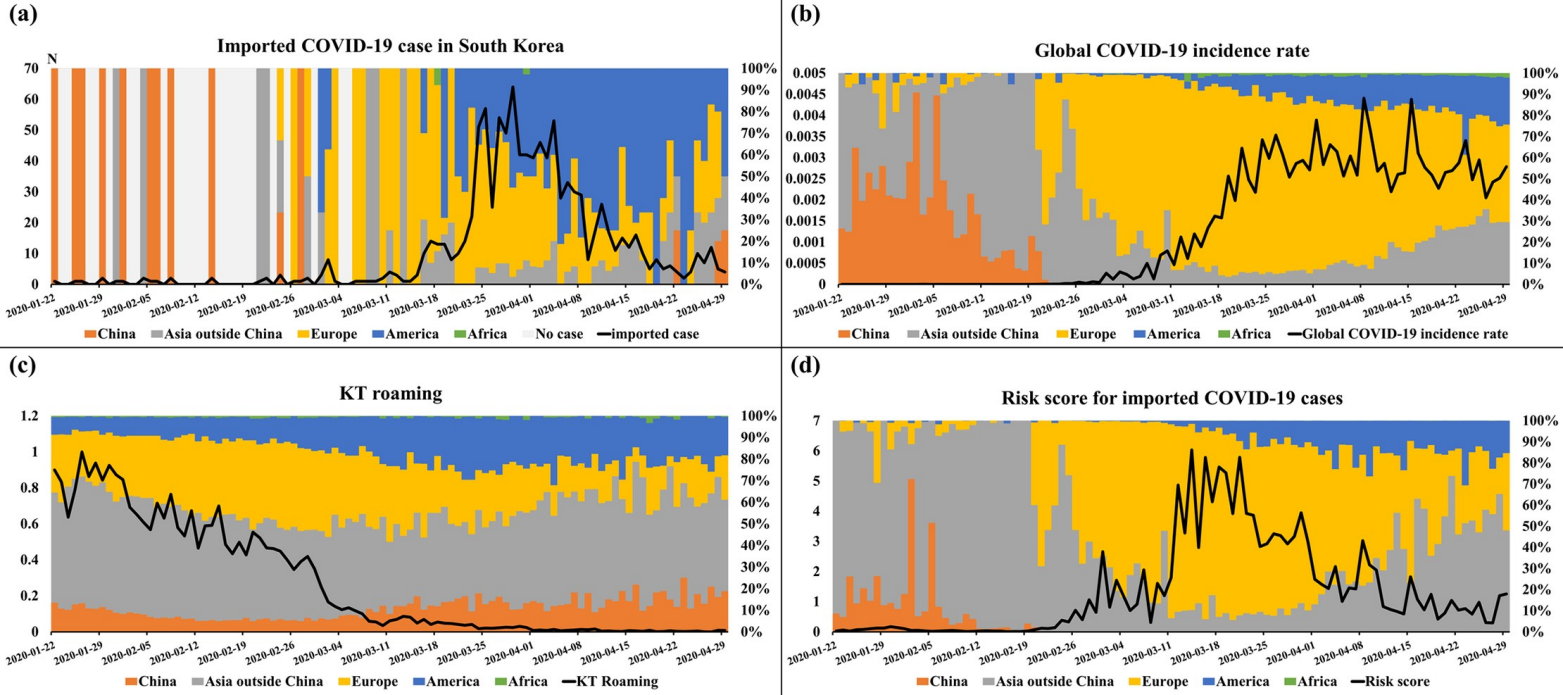
Imported COVID-19 cases in South Korea. (A) The time-series data of imported COVID-19 cases in South Korea. (B) Global COVID-19 incidence rate. (C) Korea Telecom (KT) roaming data. (D) Risk score of imported COVID-19 cases. The stacked vertical bar graph at 100% represents the ratio of daily values of countries categorized as China, Asia except China, Europe, America, and Africa. KT roaming data were converted to have a range from 0 to 1 by Min-Max Scaling.

Fig 1 shows the output variable (imported COVID-19 cases in South Korea) and the input variables, namely global COVID-19 incidence rate, KT roaming data, and the risk score from imported COVID-19 cases. Fig 1 also presents the ratio of daily values of countries by a stacked vertical bar graph at 100%; the countries are categorized as China, Asia except China, Europe, America, and Africa. In Fig 1A, the start and peak points of the imported cases are shown on January 22 and April 3. As seen in Fig 1B, the outbreak of COVID-19 began to increase in Europe and the United States from the beginning of March. Of the total confirmed cases, the proportion of confirmed cases in Asia except China and the United States gradually increased since March but decreased in Europe since April. Fig 1C shows that the time-series roaming data began to decrease gradually after January 22 because of the COVID-19 pandemic. Fig 1D shows that the risk score of the imported COVID-19 cases; the pattern of time-series for the risk score was similar to the imported COVID-19 cases in Fig 1A. Moreover, the risk score was significantly correlated with the imported COVID-19 cases, and the correlation coefficient of 0.844 with -12 days of time lag was calculated by the cross-correlation analysis. As a result, we selected 12 days-ahead forecast.

### Prediction models

The forecasting models for imported COVID-19 cases after 12 days were developed using LR and ARIMAX with the risk score. After preprocessing the 7-day simple moving average, the adjusted R-squared value of the LR model using the training set was 0.849, and the beta coefficient was 9.144 with p < 0.001. The residuals from the LR indicated the presence of autocorrelation because the result of the Durbin–Watson test was close to zero (0.205). We compared the performance of ARIMA and ARIMAX to verify the risk score, excluding autocorrelation of the dependent variable. Table 1 shows the performance of the LR, ARIMA (*p*,*d*,*q*), and ARIMAX (*p*,*d*,*q*) using the first validation set and second validation set. The best model of ARIMA with optimal parameters was ARIMA (*1*,*1*,*0*), and the best model of ARIMAX was ARIMAX (*1*,*1*,*0*) in both first validation sets selected by the RMSE. The AICs of ARIMAX using the risk score were lower than those of ARIMA models. The RMSE (6.3) of ARIMAX (*1*,*1*,*0*) was much lower than the RMSE (22.3) of ARIMA (*1*,*1*,*0*). In the first validation set, the RMSE (6.2) for the LR model was lower than that of ARIMA (*1*,*1*,*0*). Moreover, the performances of ARIMAX were better than those of ARIMA in the second validation set.

**Table 1:** Performance of the forecasting models for imported COVID-19 cases after 12 days.

Model	(p,d,q)	Training set			First validation set (3.28~4.30)				Second validation set (5.1~6.30)			
		AIC	BIC	P-value*	*R*	RMSE	MAE	MAPE	*R*	RMSE	MAE	MAPE
LR with risk score		269.0	273.0	< 0.001	0.885	6.2	5.0	56.5	0.835	4.4	3.5	32.8
ARIMA (Reference)	(1,1,0)	165.3	169.2	-	0.899	22.3	15.3	117.1	0.716	4.7	3.5	30.9
	(1,1,1)	135.3	141.1	-	0.885	96.9	40.6	186.8	0.657	5.1	4.0	39.4
	(2,1,0)	154.4	160.2	-	0.894	36.4	19.4	115.9	0.662	5.0	3.9	37.6
	(2,1,1)	137.3	145.1	-	0.879	98.3	41.6	196.5	0.667	5.0	3.7	34.1
	(2,1,2)	137.1	146.8	-	0.875	99.6	41.6	192.5	0.650	5.2	4.0	38.4
ARIMAX with risk score	(1,1,0)	99.3	105.1	0.029	0.925	6.3	5.0	49.9	0.798	4.1	2.8	22.3
	(1,1,1)	100.6	108.3	0.040	0.924	6.4	5.1	50.7	0.799	4.2	2.8	22.7
	(2,1,0)	101.5	109.3	0.026	0.925	6.3	5.0	49.9	0.798	4.1	2.8	22.3
	(2,1,1)	101.3	110.9	0.050	0.935	6.8	5.4	51.2	0.798	4.1	2.8	22.3
	(2,1,2)	104.5	115.9	0.034	0.752	14.2	8.8	74.0	0.799	4.2	2.8	22.9

p, autoregressive order; d, differencing; q, moving average order; AIC, Akaike information criterion; BIC, Bayesian information criterion; LR, linear regression; *R*, correlation coefficient; RMSE, root mean square error; MAE, mean absolute error; MAPE, mean absolute percentage error; ARIMA, autoregressive moving average; ARIMAX, ARIMA including exogenous variables.

* P-value for the risk score in LR and ARIMAX models.

**Fig 2:**
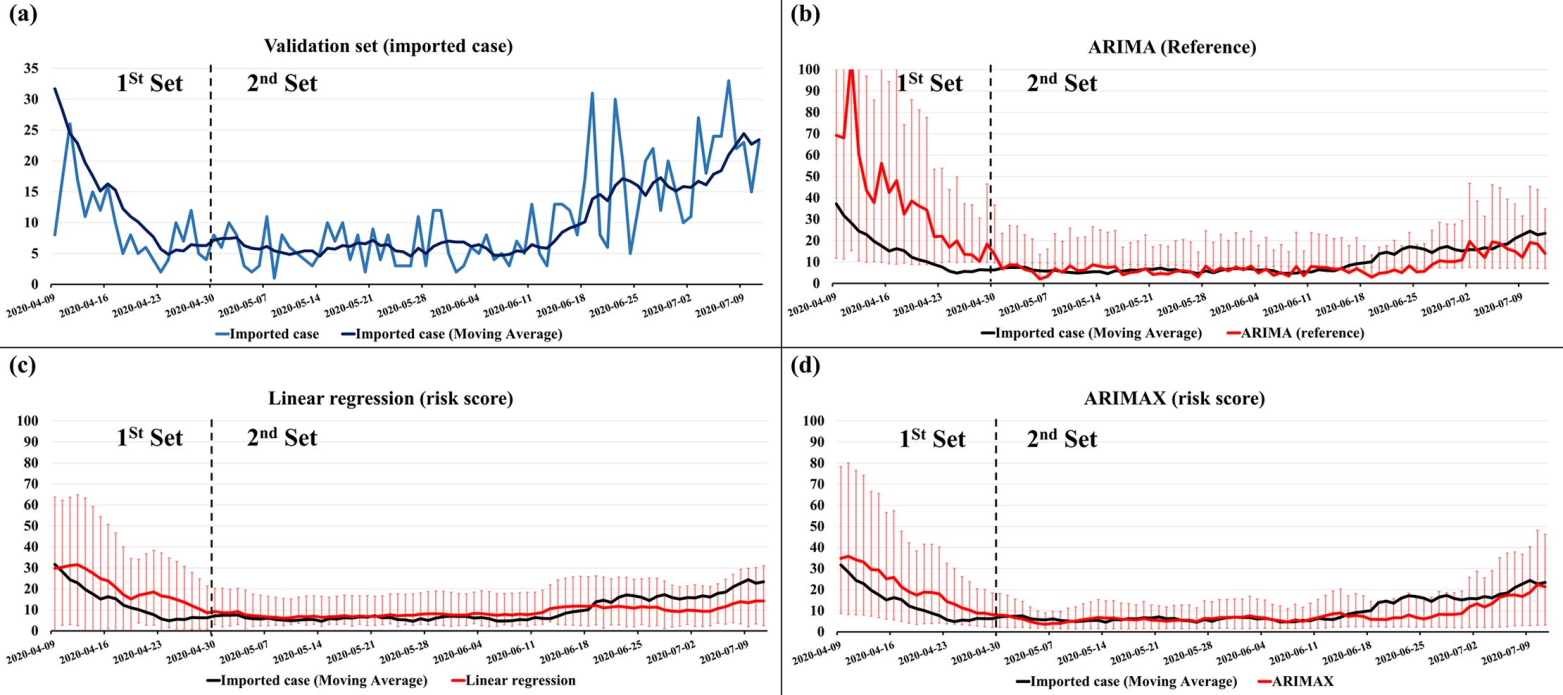
The forecast of imported COVID-19 cases after 12 days in South Korea. The forecast in both first and second validation sets is displayed. The black line denotes real imported cases in South Korea, the red line denotes the prediction values, and the red bars represent the 50% confidence intervals.

**Fig 3:**
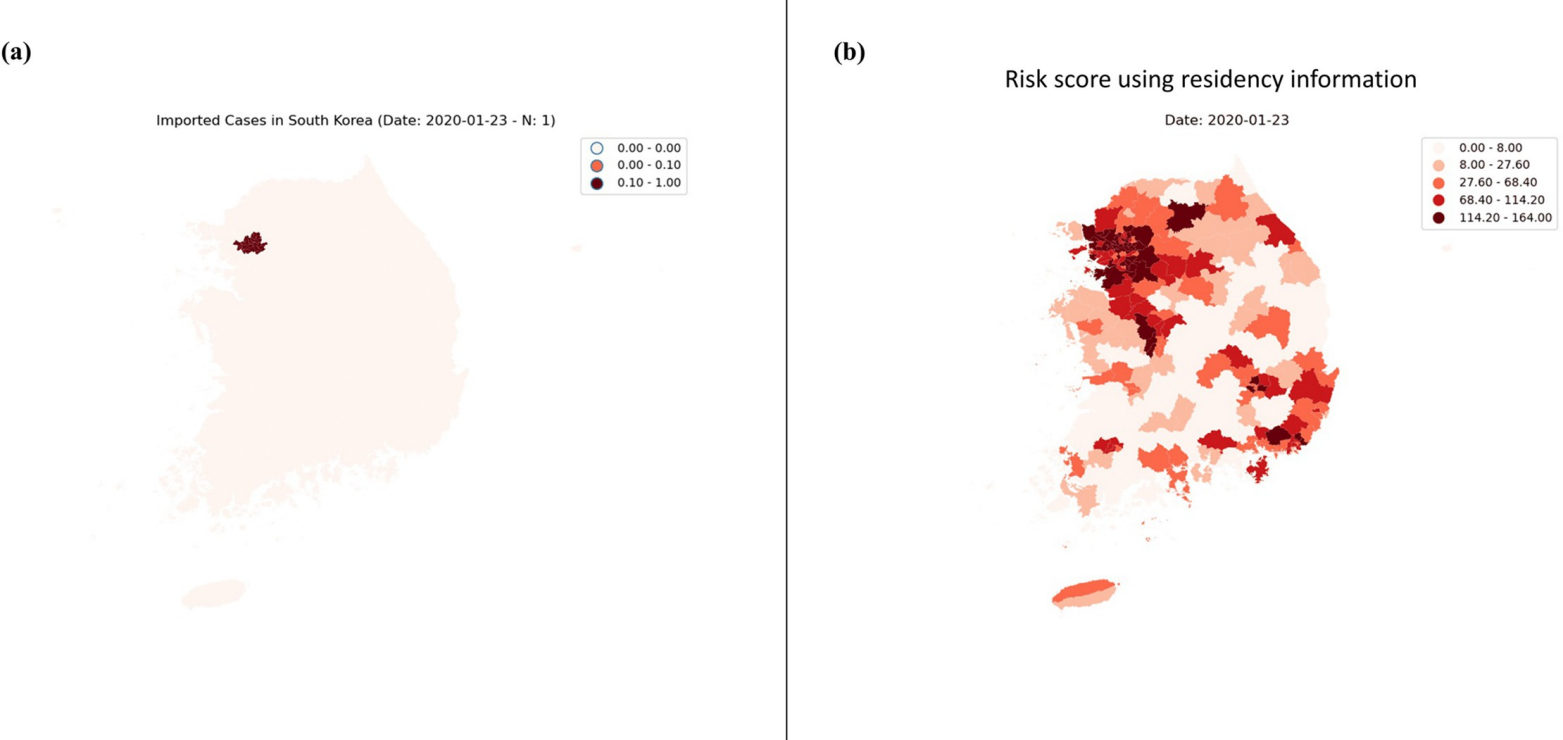
Heat maps of imported COVID-19 cases in South Korea. (A) Heat maps for the imported COVID-19 cases. (B) The risk score calculated using the roaming data. Darker shades of red indicate that more imported COVID-19 cases have been confirmed (A) and present a higher risk (B).

Fig 2 shows the forecasting results for imported COVID-19 cases after 12 days using LR, ARIMA, and ARIMAX, with 50% confidence intervals, which indicate the forecast uncertainty. Fig 3A shows heat maps for areas where the imported COVID-19 cases were confirmed, and Fig 3B shows heat maps for areas where the risk scores based on residency information of the roaming users were high. Animated S1 Fig showing the risk scores from January 23, 2020 to June 30, 2020 is attached as a supplementary file.

## Discussion

This study aimed to develop the risk score for imported COVID-19 cases in South Korea using global COVID-19 data, KT roaming data, and the OxCGRT index. Further, it attempted to validate the prediction models for daily imported cases after 12 days using the risk score. The calculated risk score was significantly correlated with the imported COVID-19 cases with a time lag of -12 days; this proves that our hypothesis was correct. Moreover, the risk score made it possible to forecast imported cases after 12 days. The performance of the ARIMAX using the risk score outperformed that of the ARIMA without the risk score.

The peak of the imported COVID-19 cases in early April seems to be the intersection between the time-series of decreasing international travel and increasing COVID-19 cases. The government of the United States announced the lockdown of New York City on March 22, and the lockdown of the United Kingdom started on March 24 [30]. With the worldwide announcement for the lockdown, Korean residents and students in other countries decided to return to South Korea [31]. Because of these reasons, the imported cases may have increased in early April. Therefore, the roaming data are correlated with the imported cases with a time lag of several days.

The time lag for the forecast was selected as 12 days by cross-correlation analysis between the risk score and imported COVID-19 cases. The potential reason for the 12-day time lag in the forecast could be related to the period between exposure to the severe acute respiratory syndrome coronavirus 2 and the confirmation of infection. Lauer et al. reported that those exposed to this virus would develop symptoms within 11.5 days [32]. Reducing international travel or enforcing government policies on COVID-19 may reduce the number of imported COVID-19 cases after 12 days rather than immediately.

In Fig 1, the time-series ratios of confirmed COVID-19 cases and imported cases grouped by country show similar trends without the time lag. From January to early February, the proportion of confirmed COVID-19 cases in China was the highest globally; therefore, the imported COVID-19 cases in South Korea mostly came from China. In early April, the proportion of imported cases from Europe was the highest because the number of confirmed cases had greatly increased in Europe. However, unlike the risk score, the ratios of COVID-19 cases grouped by country did not have a time lag of 12 days. We hypothesize that the delay between the infected time and the reported time is similar for cases confirmed in other countries and imported in South Korea. Therefore, the time-series ratios of confirmed cases grouped by country could not be used to forecast ratios of the countries for imported cases after 12 days. However, information on the COVID-19 risk of the countries for imported cases can inform roaming users of the risk depending on their travel country and itinerary. Moreover, the residency information of the high-risk roaming users may help KCDC monitor risky regions in South Korea, where domestic COVID-19 outbreaks could begin.

Wells et al. demonstrated the exportation risk from mainland China using the number of airports in the country with direct flights to and from mainland China and estimated the impact of the travel lockdown of China on COVID-19 outbreaks [33]. Since COVID-19 has been declared a pandemic, it is important to consider the disease incidence and international flight information worldwide. Wells et al. mentioned that international travelers in the presymptomatic incubation period may not be detected by respiratory symptoms during the quarantine [33]. In addition, the possibility of the transmission of COVID-19 from asymptomatic patients could make it difficult to screen for imported COVID-19 cases [34]. Therefore, predicting the probability of imported COVID-19 cases could be useful in screening suspected patients among international travelers.

To the best of our knowledge, this is the first study to forecast imported COVID-19 cases after 12 days and validate the risk score with KT roaming data. If international traveler information, such as roaming data, is available for a specific country, the risk score suggested in this study can be calculated to predict the imported COVID-19 cases for that country. However, our study has several limitations. The KT roaming data could not cover all international travel. Among those entering South Korea, there may be individuals who do not use KT services or use telecommunication providers other than KT. Furthermore, the roaming data cannot be disclosed because of the privacy laws of South Korea. Further research to develop prediction models for the domestic outbreak of COVID-19 based on the calculated risks of imported COVID-19 cases is warranted.

## Conclusions

This study demonstrated the performance of the risk score of imported COVID-19 cases using roaming data. The risk score had a negative time lag of 12 days and is eligible as an input variable for the 12 days forecast of imported COVID-19 cases in South Korea. The roaming data could help warn roaming users that they are at a high risk of contracting COVID-19 and inform the KCDC of possible high-risk areas for domestic outbreaks using the residency information of travelers returning to South Korea. Although the roaming data cannot cover all international travel, the number of international travelers could be estimated using additional data from the government and passenger airlines. Therefore, we suggest using the risk score to forecast imported COVID-19 cases after 12 days. Further, it could help the KCDC to determine resource allocation for the quarantine system.

## Supporting information

S1 Fig (PPSX)

## References

[1] Dong E, Du H, Gardner L. An interactive web-based dashboard to track COVID-19 in real time. Lancet Infect Dis 2020; 3099(20):19–20. 10.1016/S1473-3099(20)30120-1 PMC7159018 32087114

[2] KCDC. The updates on COVID-19 in Korea. Seoul, Korea: Korea Centers for Disease Control and Prevention; 2020 http://ncov.mohw.go.kr/en

[3] Gössling S, Scott D, Hall CM. Pandemics, tourism and global change: a rapid assessment of COVID-19. J Sustain Tour 2020 10.1080/09669582.2020.1758708

[4] COVID-19 infections accelerate in Germany after lockdown easing. May 11, 2020, CGTN https://newseu.cgtn.com/news/2020-05-11/COVID-19-infections-accelerate-in-Germany-after-lockdown-easing-QocbRNMXrq/index.html

[5] Tatem AJ, Huang Z, Das A, Qi Q, Roth J, Qiu Y. Air travel and vector-borne disease movement. Parasitology 2012; 139(14):1816–30. 10.1017/S0031182012000352 22444826

[6] Wesolowski A, Buckee CO, Engø-Monsen K, Metcalf CJE. Connecting Mobility to Infectious Diseases: The Promise and Limits of Mobile Phone Data. J Infect Dis 2016; 214(suppl_4):S414–20. 10.1093/infdis/jiw273 28830104 PMC5144902

[7] Wesolowski A, Metcalf CJ, Eagle N, et al Quantifying seasonal population fluxes driving rubella transmission dynamics using mobile phone data. Proc Natl Acad Sci U S A 2015; 112(35):11114–9. 10.1073/pnas.1423542112 26283349 PMC4568255

[8] Girardin F, Dillenbourg P, Nova N. Detecting air travel to survey passengers on a worldwide scale. J Locat Based Serv 2009: 3(3);210–26.

[9] IATA. May Passenger Demand Shows Slight Improvement. 1 July 2020 https://www.iata.org/en/pressroom/pr/2020-07-01-02/

[10] Hale T, Petherick A, Phillips T, Webster S. Variation in government responses to COVID-19. Blavatnik school of government working paper. 2020; 31. https://www.bsg.ox.ac.uk/research/publications/variation-government-responses-covid-19

[11] KCDC. Coronavirus Disease-19, Republic of Korea. http://ncov.mohw.go.kr/en

[12] Worldometers: COVID-19 coronavirus pandemic. https://www.worldometers.info/coronavirus/#countries

[13] Statista. Market share of mobile phone service providers based on the user number in South Korea in 2019. https://www.statista.com/statistics/700467/south-korea-mobile-phone-market-share/

[14] Lee KT, KT joint research on predicting the spread of ‘Wuhan Corona’ with AI and big data, Chosun Biz, March 24, 2020, https://biz.chosun.com/site/data/html_dir/2020/03/24/2020032400735.html

[15] Data Safe Zone, https://dsz.kdata.or.kr/svc/main/main.do

[16] Korea Data Agency, http://global.kdata.or.kr/en/kdata/

[17] Lee S, Liao Y, Seo MH, Shin Y. Sparse HP Filter: Finding Kinks in the COVID-19 Contact Rate. 2020. Available online 26 September 2020 10.1016/j.jeconom.2020.08.008 PMC7519716 33012953

[18] Choi SB, Kim J, Ahn I. Forecasting type-specific seasonal influenza after 26 weeks in the United States using influenza activities in other countries. PLoS One 2019; 14(11):e0220423 10.1371/journal.pone.0220423 31765386 PMC6876883

[19] Kongcharoen C, Kruangpradit T. Autoregressive integrated moving average with explanatory variable (ARIMAX) model for Thailand export. In 33rd International Symposium on Forecasting, South Korea, 2013; 1–8.

[20] Shojaei A, Flood I. Univariate Modeling of the Timings and Costs of Unknown Future Project Streams: A Case Study. Int J Adv Sys Meas 2018; 11:36–46.

[21] Willmott CJ, Matsuura K. Advantages of the mean absolute error (MAE) over the root mean square error (RMSE) in assessing average model performance. Clim res 2005: 30(1);79–82.

[22] Zeng Q, Wen H, Huang H, Pei X, Wong SC. A multivariate random-parameters Tobit model for analyzing highway crash rates by injury severity. Accid Anal Prev 2017; 99:184–91. 10.1016/j.aap.2016.11.018 27914307

[23] Zeng Q, Guo Q, Wong SC, Wen H, Huang H, Pei X. Jointly modeling area-level crash rates by severity: a Bayesian multivariate random-parameters spatio-temporal Tobit regression. Transportmetrica A: Transport Science 2019; 15(2):1867–84.

[24] Zeng Q, Wen H, Wong SC, Huang H, Guo Q, Pei X. Spatial joint analysis for zonal daytime and nighttime crash frequencies using a Bayesian bivariate conditional autoregressive model. J Transp Saf Secur 2020; 12(4):566–85.

[25] Burnham KP, Anderson DR. Model Selection and Multimodel Inference: A Practical Information-Theoretic Approach, 2nd edition, Springer-Verlag, New York, 2002.

[26] Wu Q, Chen F, Zhang G, Liu XC, Wang H, Bogus SM. Mixed logit model-based driver injury severity investigations in single- and multi-vehicle crashes on rural two-lane highways. Accid Anal Prev 2014; 72:105–15. 10.1016/j.aap.2014.06.014 25016459

[27] Chen F, Chen S, Ma X. Crash Frequency Modeling Using Real-Time Environmental and Traffic Data and Unbalanced Panel Data Models. Int J Environ Res Public Health 2016; 13(6):609 10.3390/ijerph13060609 27322306 PMC4924066

[28] Chen F, Chen S, Ma X. Analysis of hourly crash likelihood using unbalanced panel data mixed logit model and real-time driving environmental big data. J Safety Res 2018; 65:153–9. 10.1016/j.jsr.2018.02.010 29776524

[29] White KJ. The Durbin-Watson test for autocorrelation in nonlinear models. Rev Econ Stat 1992;370–3.

[30] S. Korea's first evacuation plane heads to China's Wuhan. Yonhap News Agency. Jan 30, 2020. https://en.yna.co.kr/view/AEN20200130000458325

[31] Over 30,000 S. Koreans return home from abroad amid coronavirus pandemic. Yonhap News Agency. May 24, 2020. https://en.yna.co.kr/view/AEN20200524002700325

[32] Lauer SA, Grantz KH, Bi Q, et al The Incubation Period of Coronavirus Disease 2019 (COVID-19) From Publicly Reported Confirmed Cases: Estimation and Application. Ann Intern Med 2020; 172(9):577–82. 10.7326/M20-0504 32150748 PMC7081172

[33] Wells CR, Sah P, Moghadas SM, et al Impact of international travel and border control measures on the global spread of the novel 2019 coronavirus outbreak. Proc Natl Acad Sci U S A 2020; 117(13):7504–9. 10.1073/pnas.2002616117 32170017 PMC7132249

[34] Gandhi M, Yokoe DS, Havlir DV. Asymptomatic Transmission, the Achilles' Heel of Current Strategies to Control Covid-19. N Engl J Med 2020; 382(22):2158–60. 10.1056/NEJMe2009758 32329972 PMC7200054

