# Supplementary figures and images for: Forecasting imported COVID-19 cases in South Korea using mobile roaming data
Source: PLoS One. 2020 Nov 4;15(11):e0241466. doi: 10.1371/journal.pone.0241466 (PMC7641397; doi:10.1371/journal.pone.0241466)

## Slide 1
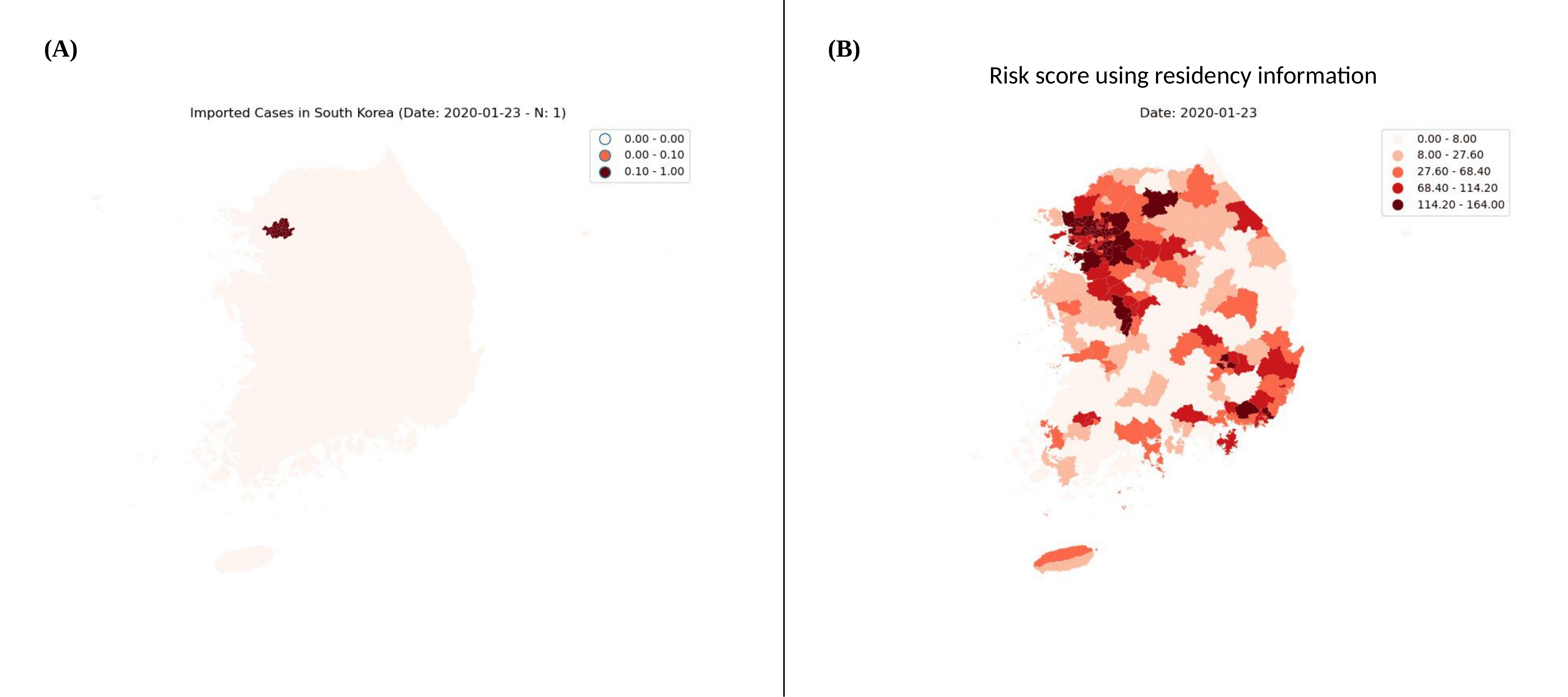

Supplement: S1 Fig — (PPSX) [file pone.0241466.s001.ppsx]
